# Rapamycin Plus Doxycycline Combination Affects Growth Arrest and Selective Autophagy-Dependent Cell Death in Breast Cancer Cells

**DOI:** 10.3390/ijms22158019

**Published:** 2021-07-27

**Authors:** Titanilla Dankó, Gábor Petővári, Dániel Sztankovics, Dorottya Moldvai, Regina Raffay, Péter Lőrincz, Tamás Visnovitz, Viktória Zsiros, Gábor Barna, Ágnes Márk, Ildikó Krencz, Anna Sebestyén

**Affiliations:** 1 1st Department of Pathology and Experimental Cancer Research, Semmelweis University, Üllői út 26, H-1085 Budapest, Hungary; tita.danko@gmail.com (T.D.); gaborpetovari@gmail.com (G.P.); sztankovics.daniel@gmail.com (D.S.); moldvai.dorottya@gmail.com (D.M.); regiraffay@gmail.com (R.R.); barna.gabor@med.semmelweis-univ.hu (G.B.); markagnes555@gmail.com (Á.M.); krencz.ildiko@gmail.com (I.K.); 2 Department of Anatomy, Cell and Developmental Biology, Eotvos Lorand University, Pázmány Péter sétány 1/c, H-1117 Budapest, Hungary; concrete05@gmail.com; 3 Department of Genetics, Cell- and Immunobiology, Semmelweis University, Nagyvárad tér 4, H-1089 Budapest, Hungary; tamas.visnovitz@gmail.com; 4 Department of Anatomy, Histology and Embryology, Semmelweis University, Tűzoltó utca 58, H-1094 Budapest, Hungary; zsiros.viktoria@med.semmelweis-univ.hu

**Keywords:** rapamycin, doxycycline, mitophagy, autophagy, cell death, tumour growth

## Abstract

Metabolic alteration is characteristic during tumour growth and therapy; however, targeting metabolic rewiring could overcome therapy resistance. mTOR hyperactivity, autophagy and other metabolic processes, including mitochondrial functions, could be targeted in breast cancer progression. We investigated the growth inhibitory mechanism of rapamycin + doxycycline treatment in human breast cancer model systems. Cell cycle and cell viability, including apoptotic and necrotic cell death, were analysed using flow cytometry, caspase activity measurements and caspase-3 immunostainings. mTOR-, autophagy-, necroptosis-related proteins and treatment-induced morphological alterations were analysed by Wes^TM^, Western blot, immunostainings and transmission electron microscopy. The rapamycin + doxycycline combination decreased tumour proliferation in about 2/3rd of the investigated cell lines. The continuous treatment reduced tumour growth significantly both in vivo and in vitro. The effect after short-term treatment was reversible; however, autophagic vacuoles and degrading mitochondria were detected simultaneously, and the presence of mitophagy was also observed after the long-term rapamycin + doxycycline combination treatment. The rapamycin + doxycycline combination did not cause apoptosis or necrosis/necroptosis, but the alterations in autophagy- and mitochondria-related protein levels (LC3-B-II/I, p62, MitoTracker, TOM20 and certain co-stainings) were correlated to autophagy induction and mitophagy, without mitochondria repopulation. Based on these results, we suggest considering inducing metabolic stress and targeting mTOR hyperactivity and mitochondrial functions in combined anti-cancer treatments.

## 1. Introduction

Regardless of the recent improvements in cancer prevention and therapy, cancer is still one of the leading causes of death worldwide. Considering the diagnosed malignancies, breast cancer is one of the most frequent neoplasms, with an elevating incidence rate [1]. Developing resistance during common and targeted therapies is in correlation with the failures of cancer care. Mammalian target of rapamycin (mTOR) complexes are in a central regulatory position in signalling networks, including metabolic rewiring in the course of tumour development. mTOR hyperactivity is characteristic for many cancers, especially in the worst cases [2,3]. Rapamycin analogues (rapalogs) were introduced into the therapy of many cancers (e.g., advanced breast cancers), but the already tested rapalogs could not achieve breakthrough success in cancer treatments [4]. The major problems are tumour evolution, dormancy/survival and late growing reactivation supported by cellular adaptation mechanisms [5,6]. As regards, targeting metabolic processes after or simultaneously with different drug administrations could be an additional option in future therapy developments [7,8].

Cancer cells have special metabolic demands for proliferation and/or survival; in addition, metabolically heterogeneous tumours can adapt to several chemotherapy-induced damages with high metabolic plasticity [6,8]. Based on this idea, ongoing trials are using metformin/phenformin, glutaminase, lipid metabolism inhibitors and autophagy inducers/inhibitors as well to inhibit activated bioenergetic mechanisms during therapy [9,10,11,12]. It could be important to describe the metabolic consequences of different drug treatments for using metabolic modulators to overcome therapy resistance.

Higher metabolic plasticity could promote late-stage tumour cell survival and relapsing tumour and metastasis formation in patients. Most targeted therapies (including EGFR inhibitors or other multi-kinase inhibitors) affect mTOR kinase activity and the related metabolic events; similarly to mTOR inhibitors, these decrease cellular glycolytic activity, inhibit glutaminolysis and shift to lipid oxidation instead of affecting lipid synthesis directly or reducing protein synthesis of several metabolic enzymes [3,13,14]. These changes can reduce tumour cell proliferation but also help to turn a more resistant dormant state (extracellular environment independent) and provide minimal energy consumption to promote cellular survival.

Tumour growth, tumour mass reductions and tumour-free survival are the main goals during cancer treatments, which could have both cytotoxic and cytostatic effects [15]. After many drug administrations, which inhibit tumour cell proliferation, the surviving cells undergo autophagy as a transient bioenergetic adaptation mechanism. In addition, a number of regulated cell death mechanisms have been described in the early or late effects of autophagy-inducing treatments. The regulatory overlap between cell death mechanisms discriminates three major modes: Type I—apoptosis, Type II—autophagy-mediated cell death and Type III—necrosis/necroptosis, with many different forms discovered in recent years [16].

Apoptosis is orchestrated by extrinsic or intrinsic pathways and associated with caspase-mediated cellular cleavage into apoptotic bodies, while necroptosis is a special form of cell death followed by organelle swelling. Autophagy delivers cellular components from the cytoplasm to autophagosomes for degradation. Certain findings highlight that cells with defected autophagy could promote malignant processes as a result of accumulating abnormal or toxic elements. On the other hand, autophagy could also be an escape mechanism for cells in stressed conditions. While apoptosis, necrosis or necroptosis are originally direct cell death forms, autophagy is rather identified as a temporary survival mechanism. Moreover, the border between autophagy-related and other types of cell death is not sharp; therefore, the role of autophagy is mainly context-dependent [17,18,19]. This makes the role of autophagy in cell death very difficult to distinguish. mTOR inhibitors induce autophagy which is accompanied by increasing autophagosomes, and in addition, chronic treatment can lead to cell death in cancers, as well. It is also well-known that autophagy regulation is under the control of available nutrients and mTOR signals [20]. Based on these, mTORC1 inactivation could be involved in both autophagy-dependent survival and cell death in cancer progression [21]. However, the autophagy-dependent cell death mechanism is less well characterised in tumour biology.

The induced reversible anti-proliferative, anti-tumour growth effects and sensitisation of different chemotherapeutics after rapalog treatment were described in our previous in vitro mTOR inhibitor studies [22,23,24].

Certain antibiotics, including doxycycline, have an off-target effect on the energy generating organelles in human cells. Other recent studies demonstrated that these antibiotics—which target mitochondrial biogenesis in common bacterial infections—could have potential anti-cancer effects. Regarding this function, anti-microbial medications point out that applying these drugs in combination with conventional or targeted therapies could have a potential benefit with less toxic side-effects for cancer patients in clinical practice [25,26,27]. Since the tetracycline analogue doxycycline was previously found to synergise with rapamycin or temozolomide in our glioma studies [24], we intended to investigate rapamycin and doxycycline treatments in different solid tumour cell lines and study the cellular mechanisms of the tumour growth inhibitory effects in our in vitro and in vivo breast cancer cell line models.

Our results demonstrated that: (a) rapamycin + doxycycline combination inhibited the proliferation significantly in 2/3rds of the studied cells in vitro; (b) the long-term in vitro and in vivo treatments were highly toxic for the studied breast cancer cells; (c) however, the short-term treatment was reversible, the continuous long-term rapamycin + doxycycline combination induced selective autophagy-dependent cell death and significant anti-tumour effects.

## 2. Results

### 2.1. Rapamycin + Doxycycline Combination Inhibits Cell Proliferation in Human Breast Cancer Cells

**Figure 1:**
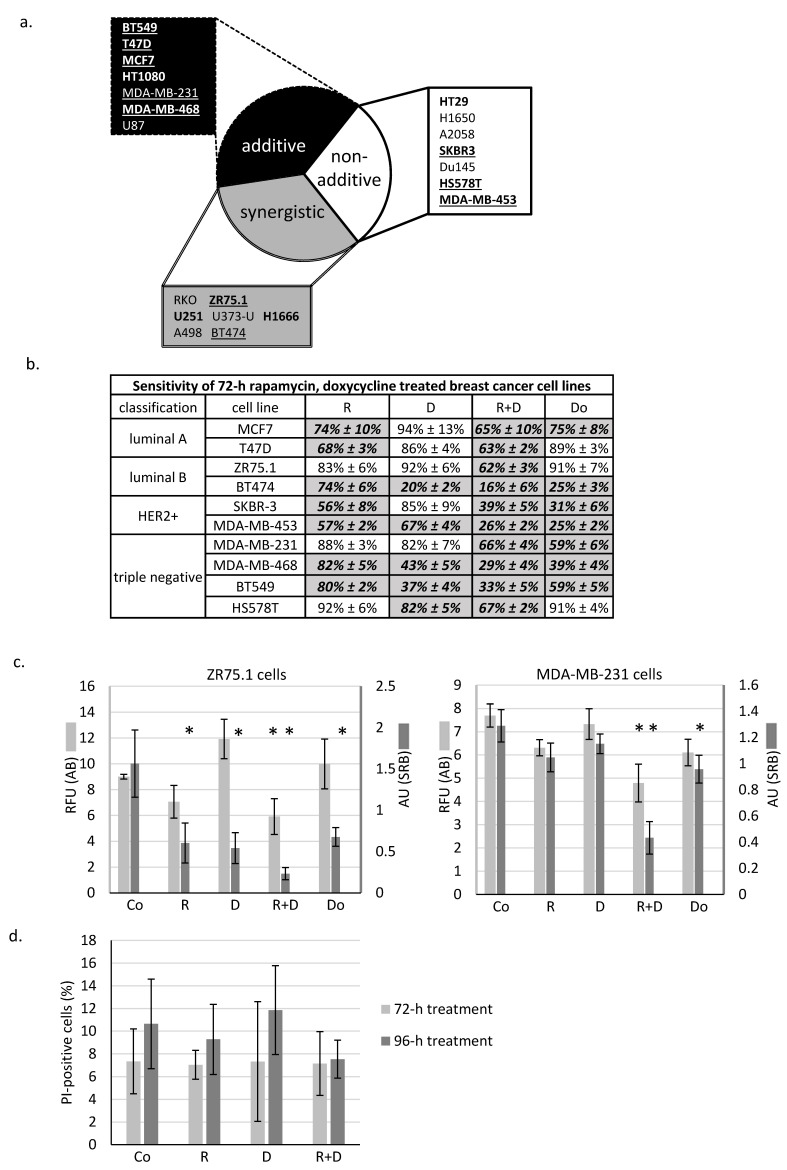
In vitro rapamycin and doxycycline combinations have significant anti-tumour growth effects in the majority of the studied cancer cell lines. (**a**) In vitro additive and synergistic anti-proliferative effects of rapamycin and doxycycline in 2/3rd of studied cells. Based on proliferation data, the effects of combined treatments could be divided into three categories: (**a**) additive; (**b**) non-additive and (**c**) synergistic. Underlined: human breast cancer cell lines, bold: proliferation inhibition >50%. The additive and synergistic effects of rapamycin and doxycycline combination were calculated using combination index. (**b**) The effects of 72-h-treatments of the 10 studied breast cancer cell lines (detected with Alamar blue assay). Cell proliferation was given in control % (mean ± SD). All data represent mean ± SD. Bold fonts with italics and grey background: *p* ≤ 0.05 (**c**) Alamar blue (AB) and sulforhodamine B (SRB) assays monitored the in vitro cell growth in ZR75.1 and MDA-MB-231 cell cultures after 72-h treatment. Data show either relative fluorescence unit (RFU; AB assay) or absorbance unit (AU; SRB test). All data represent mean ± SD. * *p* ≤ 0.05 (d) Necrosis was not detectable after rapamycin and doxycycline treatment. Flow cytometry analysis of propidium iodide (PI) stained cells in 72-h and 96-h-treated ZR75.1 cells in vitro. Results were given in control %. (Co—control; R—rapamycin, 50 ng/mL; D—doxycycline, 10 µM; R+D—rapamycin + doxycycline combination; Do—doxorubicin, 50 ng/mL).

As previously described in many different in vitro cell cultures, rapamycin has a moderate decreasing effect on tumour growth in monotherapy; however, additional anti-metabolic treatments could significantly enhance this impact. In the presented work, the effect of doxycycline antibiotic combined with rapamycin was analysed in different solid tumour-derived cell lines. We found that the rapamycin + doxycycline combination could decrease the cell proliferation capacity in either an additive or synergistic manner in about 2/3rds of the investigated cell lines (Figure 1a). In further experiments, we focused on human breast cancer cell lines. Some differences in treatment response could be observed in the ten studied breast cancer cell lines (Figure 1b); however, we could detect significant growth inhibition after the studied combination in almost all cells. Based on the proliferation results obtained, we selected two cell lines from different subtypes for additional investigations.

We observed the same tendentious proliferation inhibition using either the Alamar blue (AB) assay or sulforhodamine B (SRB) test. Accordingly, in the case of ZR75.1 (luminal B) and MDA-MB-231 (triple-negative) cell lines, the rapamycin + doxycycline combination had a more significant cell growth inhibitory effect than rapamycin or doxycycline monotherapies alone. As a consequence of combination therapy, the rate of the detected inhibition resulted in synergistic growth inhibitory effects in ZR75.1 and additive inhibition in MDA-MB-231 cells (Figure 1c). Additionally, the studied cell lines had different sensitivity rates to doxorubicin (a chemotherapeutic agent) treatment. Comparing its effect with the proliferation inhibition caused by rapamycin + doxycycline, we observed that the combinational treatment had either the same or even higher proliferation reducing effects than the chemotherapeutic drug in mono-treatment (Figure 1b,c).

### 2.2. Rapamycin + Doxycycline Combination Did Not Induce Apoptosis or Necrosis after 72-h or 96-h Treatments

**Figure 2:**
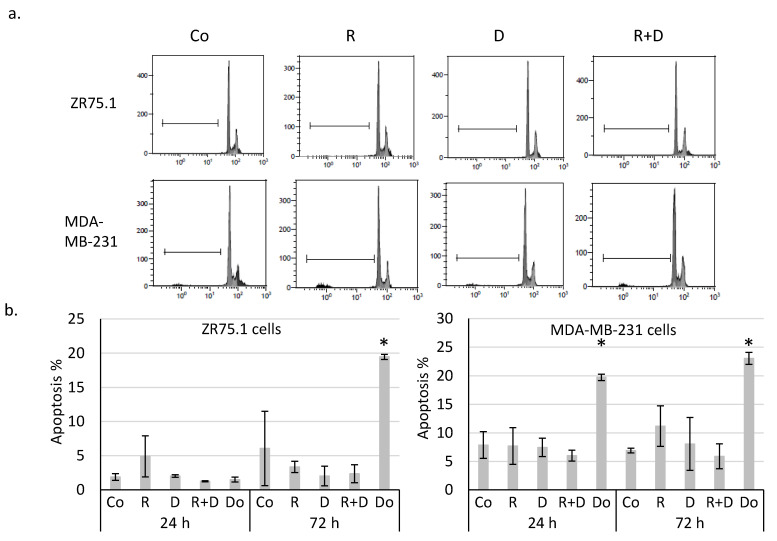

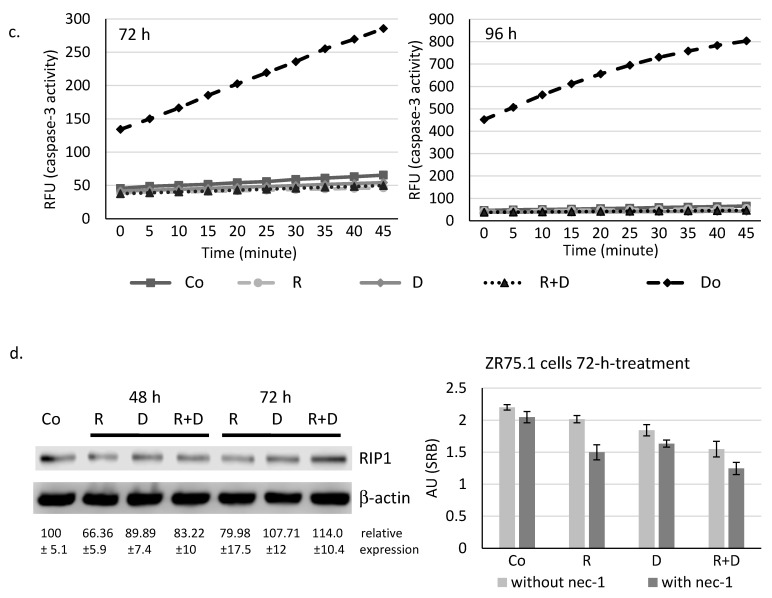
Rapamycin and doxycycline in vitro treatments do not induce apoptosis or necroptosis in 72-h-treated ZR75.1 and MDA-MB-231 cells. (**a**) Flow cytometry analyses of apoptotic cells (fixed, alkaline extracted and PI stained). Linear bar shows the apoptotic subG1 cell fraction. (**b**) The detected % of apoptotic cells in 24-and 72-h-treated ZR75.1 cells and MDA-MB-231 in vitro. * *p* ≤ 0.05 (**c**) Caspase-3 activity was not increased in vitro in 72-h or 96-h rapamycin + doxycycline-treated ZR75.1 cells. Caspase-3 activity was measured by Z-DEVD-AMC, data show relative fluorescence unit (RFU), doxorubicin treatment was the positive control in these measurements. (**d**) The sign of necroptosis, the increase of RIP1 expression was not detected in 72-h-treated ZR75.1 cells. Western blot analysis of RIP1 after 48-h and 72-h rapamycin and doxycycline treatments (left panel). Nor necrostatin-1 (nec-1) nor Ac-DEVD-CHO (caspase-3 inhibitor) could inhibit the anti-proliferative effects rapamycin + doxycycline in 72-h-treated ZR75.1 cells in vitro—data of Alamar blue assay represent relative fluorescence unit (RFU) (right panel). (Co—control; R—rapamycin, 50 ng/mL; D—doxycycline, 10 µM; R+D—rapamycin + doxycycline combination; Do—doxorubicin, 50 ng/mL, nec-1—50 µM; Ac-DEVD-CHO—1 µM).

Determining whether the observed ~40% proliferation inhibition initiated by rapamycin and doxycycline combination was caused by necrosis, ZR75.1 cells were stained with propidium iodide (PI), and the ratio of the necrotic cell population was analysed by flow cytometry. According to our findings, there was no significant difference among the amount of PI-positive cells since there was no induced necrosis in the cell cultures after 72-h (Figure 1d) and 96-h treatments. After fixation and alkaline extraction of cleaved DNA, apoptosis induction could not be detected in either 24-h or 72-h-treated ZR75.1 and MDA-MB-231 cells by flow cytometry measurements (Figure 2a,b). The ZR75.1 cell line was selected for further cell death-related in vitro experiments. To confirm the previous findings and investigate whether apoptosis could be induced in our in vitro model system, caspase-3 activity measurement was carried out using fluorogenic Z-DEVD-AMC. Accordingly, increasing caspase-3 activity, cleaved Z-DEVD-AMC was only detected after doxorubicin treatment (for 72 and 96 h). In contrast, neither rapamycin/doxycycline in monotherapy, nor the combination treatment enhanced apoptotic cell death in ZR75.1 cell line (Figure 2c). In parallel, the applied Ac-DEVD-CHO addition for caspase inhibition did not have any effect in treated ZR75.1 cells (Figure 2d). As no significant elevation in the expression of RIP1 could be detected at protein level (Western blot), we found that neither rapamycin and doxycycline mono-treatments nor the combination induced necroptosis in 48 or 72 h treated ZR75.1 cells. This observation was also confirmed with the use of necrostatin-1 (nec-1). Nec-1 treatment (72 h) could not significantly inhibit the anti-proliferative effects of the used treatments; however, the necroptosis inhibitor had a slight inhibitory effect on cell proliferation. As a result, we showed that the observed growth arrest was not a consequence of necroptosis in our experiments (Figure 2d).

### 2.3. The Effect of Long-Term Treatment and Treatment Withdrawal in ZR75.1 Breast Cancer Cells In Vitro and In Vivo

**Figure 3:**
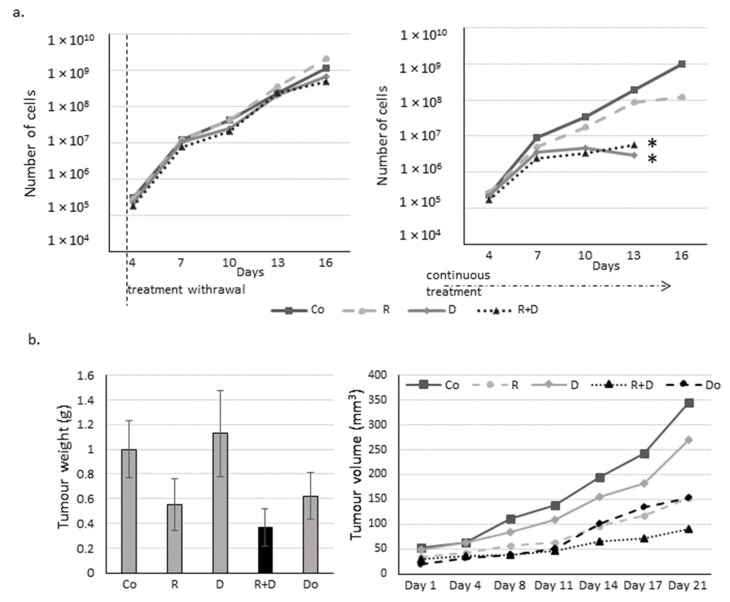
Long-term effect of rapamycin/Rapamune + doxycycline in ZR75.1 cells. (**a**) The growth curves of rapamycin and doxycycline treatments in ZR75.1 cells in vitro. The experiment was performed for 16 days. After a 72-h pre-treatment with rapamycin and doxycycline, treatments were either withdrawn (left panel) or continued as a long-term treatment (right panel) for an additional 12 days. Each nod labels a subculture. Results represent cumulative cell numbers. (Co—control; R—rapamycin, 50 ng/mL; D—doxycycline, 10 µM; R+D—rapamycin + doxycycline combination) * labels the stopped cell growth (no more viable cells). (**b**) The measured tumour weights (left panel) and the growth curve (right panel) of the ZR75.1 xenografts after 21-day treatments. Rapamune + doxycycline significantly reduced the tumour growth (black column). (Co—control; R—rapamycin/Rapamune 3 mg/kg; D—doxycycline 5 mg/kg; R+D—rapamycin/Rapamune + doxycycline combination; Do—doxorubicin, 2 mg/kg).

To reveal whether the proliferation inhibitory effects of rapamycin and doxycycline monotherapy or their combination is reversible or causes irreversible changes for maintaining cell proliferation, we performed long-term experiments using cell cultures in vitro and in vivo in xenograft models. The proliferation rate of cells treated only 72 h was restored after drug withdrawal, and the previously detected lag only remained for the next ~ two weeks after treatments. However, in the case of the continuous treatment course with doxycycline or rapamycin + doxycycline addition, cell growth was markedly declined (Figure 3a); and finally, after about a two-week incubation period, the cell proliferation stopped completely and the treated in vitro cell cultures were no longer viable. The significant tumour growth decreasing effects of rapamycin and rapamycin + doxycycline were also observed in in vivo models after a three-week treatment. In contrast, doxycycline monotherapy did not decrease tumour growth as when it was detected in in vitro treatment. The rapamycin + doxycycline combination was more effective than traditional chemotherapy—doxorubicin—or the used rapamycin monotherapy in the in vivo experiments (Figure 3b). The withdrawal and further in vivo treatments were also tested, which showed that continuous treatment could cause significant survival benefits in vivo. However, the combination did not kill all tumour cells; the untreated tumour started to grow again (Figure S1). We found that the in vivo effect could be reversed the surviving tumour cells could start to grow again.

### 2.4. Induced Autophagy and Mitophagy in Rapamycin + Doxycycline Treatments

**Figure 4:**
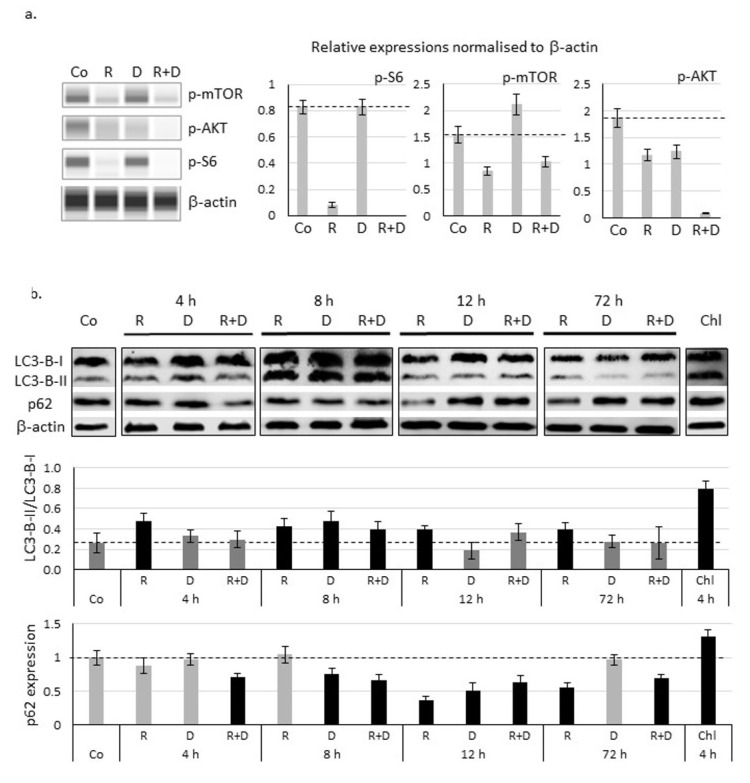
Decreased mTOR activity and activation of autophagy after in vitro rapamycin and doxycycline treatment in ZR75.1 cell line. (**a**) Decreased expressions of mTOR activity-related proteins were assessed by Wes^TM^ Simple technique (left panel). β-actin was used as loading control. Densitometric analysis of the studied proteins was presented in the right panel. (**b**) Time-dependent protein level changes of LC3-B-I/II and p62 detected with Western blot confirmed the autophagy induction. Protein level was normalised to β-actin. Chloroquine (Chl) was the positive control in this experiment. The results LC3-B-II/I ratio and relative expression of p62 based on densitometric analysis of the studied proteins were presented in the middle and lower panels. Dashed lines show the obtained level of control values. Data represent mean ± SD. (Co—control; R—rapamycin, 50 ng/mL; D—doxycycline, 10 µM; R+D—rapamycin + doxycycline combination; Chl—chloroquine, 50 µM).

The potential autophagy activation was studied in ZR75.1 cells in vitro. Decreased amounts of mTOR activity-related proteins (p-mTOR, p-AKT and p-S6) were confirmed in ZR75.1 cells treated with rapamycin or rapamycin + doxycycline combination for 72 h. In contrast, doxycycline monotherapy did not reduce p-mTOR and p-S6 proteins, but we could detect the slight decrease in p-Ser473-AKT levels compared to control (Figure 4a; Figure S2).

Time-dependent differences in the ratio of LC3-B-II and LC3-B-I (elevation) proteins and the reverse p62 decrease confirmed that autophagy induction could be observed in the early period of rapamycin and combined treatments (Figure 4b; Figure S2). Based on these, after 72-h treatments, rapamycin + doxycycline stimulated autophagy flux.

**Figure 5:**
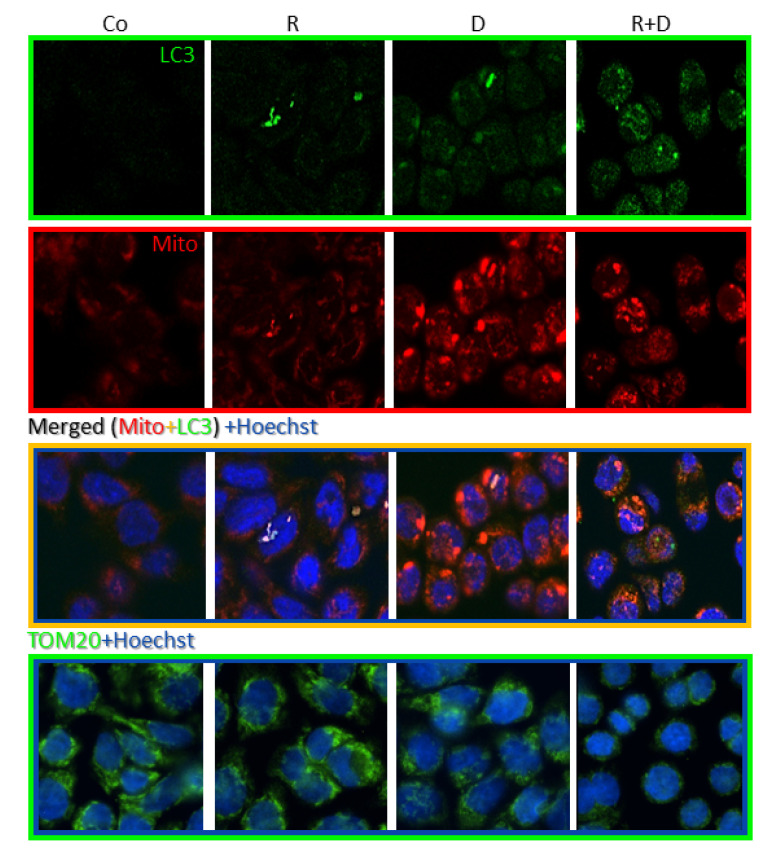
Fluorescence stainings of mitochondria and autophagy markers, and cellular adaptation to 72-h rapamycin and doxycycline treatments in ZR75.1 cell line in vitro. Fluorescence images show the staining intensity and cellular distribution of MitoTracker (red), LC3 (green) and their co-localisation (as merged) or TOM20 (green) in treated ZR75.1 cells after immunostainings using confocal microscopy. Hoechst dye was used to counterstain nuclei (blue); (magnification 63×). (Co—control; R—rapamycin, 50 ng/mL; D—doxycycline, 10 µM; R+D—rapamycin + doxycycline combination).

Fluorescence immunostaining with LC3 antibody showed that rapamycin and doxycycline monotherapy slightly mediated, while the rapamycin + doxycycline combination treatment remarkably mediated the accumulation of autophagosomes in vitro. To study the mitochondria of cells, we used MitoTracker and TOM20 labellings. Accordingly, the highest staining intensity of MitoTracker showed an unexpected morphology of the potentially clamped organelles, which indicated mitochondrial malfunction in doxycycline-treated cells. The co-localisation result of LC3 and MitoTracker co-staining and the disappearance of TOM20 protein evidenced that mitophagy could be characteristic for rapamycin + doxycycline combination treatment (Figure 5).

**Figure 6:**
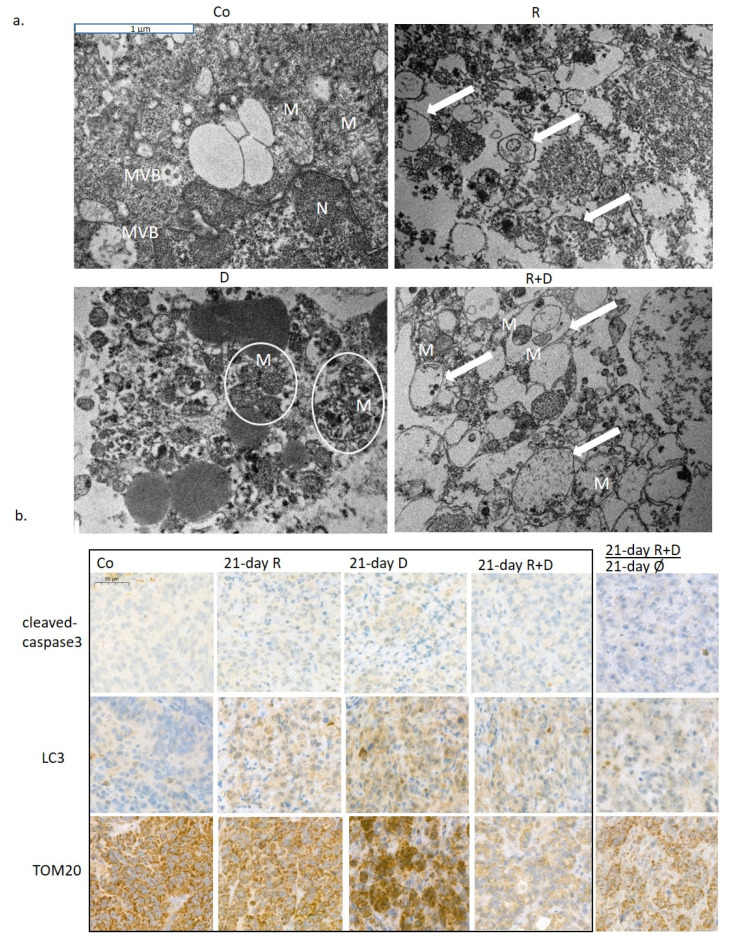
The in vivo effects of long-term rapamycin and doxycycline treatment in ZR75.1-derived xenografts. (**a**) 3-week-treated tumour xenografts were processed for transmission electron microscopy. Intact mitochondria were presented in untreated tumour sections, the number of autophagosomes was increased after Rapamune treatment; accompanied, and damaged mitochondria could be observed in doxycycline-treated samples. After administrating combined Rapamune and doxycycline treatment, collapsed mitochondria appeared in autophagic vacuoles. Mitochondria (M; clamped mitochondria were framed with white ellipsoids), autophagic vacuoles (arrow); nucleus (N); multivesicular bodies (MVB). (magnification: 20,000×). (**b**) Immunohistochemistry staining for analysing the expression of cleaved caspase-3 (apoptosis marker), LC3 (both LC3-B-I and II autophagosome proteins) and TOM20 (mitochondrial marker) in ZR75.1 xenografts. Treatments were administered for 21 days; after 21-day therapy, the drug withdrawal was also tested for an additional 21 days ($\frac{21\;days\;R+D}{+21\;days\;Ø}$). Images show representative immunostainings. DAB substrate was used for developing immunoreaction (brown), and specimens were counterstained with haematoxylin. Scale bar in the first photo—labels 50 µm. (Co—control; R—Rapamune 3 mg/kg; D—doxycycline 5 mg/kg; R+D—Rapamune + doxycycline combination; Ø no treatment).

Investigating the ultrastructure of treatment-induced morphological alterations of organelles, transmission electron microscopic analysis of ZR75.1 xenograft tumours was performed. Rapamune stimulated the accumulation of autophagic vacuoles, indicating the ongoing autophagy processes. Shrunken and impaired mitochondrial structures were characteristic for doxycycline-treated tumours; moreover, these degraded organelles were aggregated and accompanied together. Autophagic vacuoles and degrading mitochondria were present simultaneously in tumours treated with rapamycin + doxycycline combination. Moreover, combination therapies that degraded mitochondria were sequestered by autophagosomes. These results also confirmed the presence of mitophagy, a consequently occurring selective autophagy process, which could turn to cell death in long-term in vitro and three-week in vivo combination treatments (Figure 6a; Figure S4). The evaluation of the immunohistochemical stainings of ZR75.1 xenograft tumours also demonstrated that the mechanism of cell death in our experimental system was in correlation with induced selective autophagic processes in contrast to apoptosis induction after Rapamune + doxycycline treatments. In xenograft tumours, there was no increase in the number of cleaved caspase-3 positive cells. In parallel, the LC3 level was elevated after Rapamune, doxycycline and combination treatments (Figure 6b). TOM20 staining confirmed the in vitro detected disruption of mitochondria—the staining almost disappeared in the tissues after three-week combination therapy. Moreover, TOM20 and LC3 stained 21-day-treated + 21-day-untreated tumours showed the potential restoration of mitochondrial functions without continuous treatment at the tissue level, as well. These results call our attention to the high metabolic plasticity, adaptation capacity of the studied ZR75.1 human breast cancer cells, as we suggested previously in our metabolic characterisation studies [28]. Moreover, our in vivo long-term Rapamune + doxycycline treatment results in significant tumour growth inhibition and survival benefits, which highlight the importance of the studied anti-metabolic treatment combinations.

## 3. Discussion

Our results highlight that simultaneous inhibition of different metabolic pathways could dramatically reduce tumour growth both in vitro and in vivo. mTOR inhibitor therapy inhibits glycolytic pathways and has additional effects, e.g., decreasing glutaminolysis and lipid metabolism, as well [3,29]. Combining this with mitochondrial function inhibitor, the resulted wide-spectrum metabolic inhibition could stop the adaptation possibilities in high-energy demand cell growth processes in tumour growth. Our results from the rapamycin + doxycycline combination experiments supported this observation. Accordingly, we detected a more than 50% reduction in cell growth in 2/3rd of the studied solid tumour-derived cell lines. Combination therapies are the main pillars of several cancer therapies, and it is well-known that every therapy has some metabolic consequences in tumour cells, the whole organism and the patients [30,31]. There are several mTOR inhibitor combination studies and clinical trials using these concepts with more or less success [32,33,34,35]. However, the metabolic consequences and the mechanism of tumour growth inhibition are described in these cases.

It has been described in many studies that mTOR inhibitors influence cell proliferation and drug sensitivity which could vary in a cell type-dependent manner. Tumour growth could be reloaded by the surviving cells since the inhibitory effect of the agents is rather cytostatic than cytotoxic [15,36]. In addition, there is growing evidence that combining mTOR inhibitors with chemotherapeutic agents (e.g., cisplatin, etc.) could have additional benefits [36,37,38,39], especially in breast cancers, which are principal models in our experiments.

There are increasing data about metabolic rewiring and alterations in cellular metabolism after different chemotherapeutic treatments. It has been observed that the administered treatments could modulate cellular mechanisms, including alterations in the activation of autophagy, oxidative phosphorylation (OXPHOS) or Warburg reverse Warburg effects cell type and treatment dependently [40,41]. For instance, doxorubicin causes DNA damage and related apoptosis; simultaneously, it has metabolism-interfering effects. To target, such alterations in combination with metformin could help to break through therapy resistance [42]. An alternative approach could be the application of other inhibitors in combinations targeting OXPHOS or glycolysis, which could also help to sensitise different advanced breast cancers [42,43,44].

Targeting mitochondrial bioenergetics as a part of metabolic adaptation has previously emerged in many different publications in correlation with dormant, stem-cell-like phenotype and TCA activation [12]. Certain results show that several antibiotics could have an effective inhibitory function on dormant breast cancer cell survival [45], in correlation with their metabolic activity. A novel term, mitocans, was introduced for defining drugs affecting mitochondrial processes with anti-tumoural effects [46]. Rapamycin + doxycycline combination was tested only in the TSC2 KO kidney tumour model [47], and we examined this among other rapalog combinations in glioma models [24]. In the present work, we confirmed the tumour growth inhibitory effects of the used rapamycin + doxycycline in many different carcinomas. We first performed a detailed analysis on the anti-tumoural effect of rapamycin + doxycycline combination in human breast cancer models. Based on clinical data, the occasional therapeutic use of antibiotics (e.g., doxycycline) during breast cancer care increase the survival period of patients [48]. In contrast, other studies related to microbiomes highlight how antibiotics reduce diversity which could have a negative impact on cancer progression [49]. Our in vitro and in vivo results strengthen the significance of the (continuous/periodic) antibiotic administration. This could be an additional therapeutic option, but the side-effects and survival benefits need to be considered.

The detected anti-tumoural effect was characterised by inhibited proliferation in parallel with the decreased mTOR activity. p-mTOR, p-S6 levels were decreased, and p-AKT was moderately lowered as it was expected after rapamycin addition. Furthermore, the doxycycline combination practically eliminated both mTORC1 and two activities (there were no detectable p-S6 and p-AKT in the samples by Wes^TM^ Simple). However, doxycycline in monotherapy has no significant influence on mTOR activity. These alterations were in correlation with the detected increasing number of autophagy vacuoles in all long-term in vivo treatments, but mitochondrial alterations were detected only in cells after doxycycline mono- or combined administrations. The effects could turn to cell death, especially in combinations, where the continuous treatment resulted in the die out of cell cultures. Neither activation of apoptotic caspases nor membrane permeabilisation-mediated necrosis or necroptosis were detected in vitro after 72 h or 96 h. Rapamycin combination with many different drugs could have such increased mTOR inhibitory and anti-tumour effects stimulating different types of cell death mechanisms [39].

The role of autophagy is controversial in cancer development. Certain findings highlight that autophagy could be an escape/survival mechanism for cells in stress conditions (e.g., nutrient deprivation, pharmacological treatments) [50]. In addition, long-term starvation and induced autophagy could turn to autophagy-dependent cell death mechanisms [51]. There are several different criteria that help to recognise the characteristic of autophagy-dependent cell death. In accordance with these, we detected certain features in our in vitro and in vivo models: accumulation of autophagy vacuoles in dying cells without any sign of apoptosis or necrosis; dynamic alterations in LC3-II and p62 or other characteristic autophagy flux protein levels; response to pathophysiological starvation stimuli (such as mTOR inhibition). Further morphological alterations were also described in correlation to autophagy-dependent cell death mechanisms, including cytoplasmic organelle depletion, mitochondria and ER disintegrations [17,19,52]. In our studies, depleted mitochondrial mass was observed in autolysosomes as a sign of mitochondrion degradation (mitophagy), which could not prevent tumour growth inhibition and cell death in our in vivo combined treatments. However, it needs to be mentioned that short-term treatment results and the in vivo drug withdrawal showed a successful metabolic treatment combination that requires continuous treatment administration. To reach complete remission and elimination of all tumour cells in vivo, more aggressive treatments and further combinations are needed. In addition, fragmented, dead cells were characteristic for the analysed shrunken tumour tissues. Similarly to this metabolic stress-induced inhibition, several bioenergetic mechanisms could block the metabolic adaptation and activate mitophagy in other published experiments [53]. In our cases, the inhibited mitochondria biogenesis by the used antibiotic treatments caused a metabolic collapse (catastrophe) of cells that could not switch to OXPHOS because mitochondria could not be repopulated (blocked mitochondrial biogenesis). It was discussed in many tumours—tumour type dependently—that degradation of mitochondria and the recycling of metabolic precursors by autophagy and mitophagy can protect against cellular death [54,55]. However, the inhibited regeneration of a mitochondrial mass caused significant tumour growth inhibition in vivo and cell culture collapse in vitro in our long-term combination treatments. Doxycycline, as a mitochondrial protein translation inhibitor, combined with OXPHOS, forcing other treatments had similar toxic effects described by other researchers in mammosphere cultures of OXPHOS-dependent breast cancer stem cells [45].

The regulation of the number and quality of mitochondria is complex and is triggered by hypoxia, metabolic stress and mitochondrial depolarisation involving fusion/fission, mitobiogenesis and mitophagy. In addition, mTOR inactivation and lowered AMPK activation can directly activate mitophagy [56]. However, non-selective autophagy, including mitophagy, is complex, and its role varies in a tumour-type and stage-dependent manner [57,58,59]. In our recent study, the detected mitophagy could be a special form of cell death when metabolically collapsed cells with degrading damaged mitochondria turn to cell death in case mitochondrial regeneration cannot be achieved [60].

**Figure 7:**
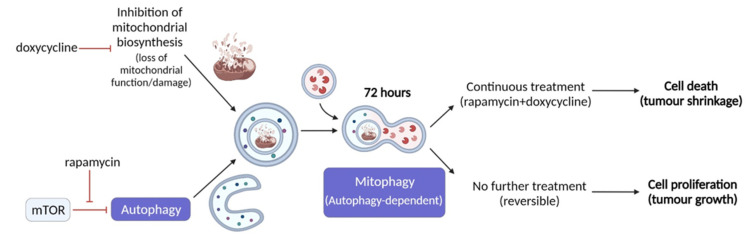
Rapamycin + doxycycline induced autophagy-dependent mitophagy in ZR75.1 breast carcinoma cells. The schematic overview illustrates the mechanism of action of rapamycin + doxycycline combination treatment. Based on our results, rapamycin + doxycycline treatment induces autophagy and consequent fusion of autophagosomes and mitochondria after 72-h treatment in vitro indicating mitophagy and autolysomal degradation. Additionally, tumour shrinkage could also be observed during a 3-week period of continuous treatment in vivo, while treatment withdrawal could lead to the recovery of cell proliferation and tumour growth.

Ours and others’ findings on the role of therapy-induced selective autophagy in cancer progression suggest that a novel strategy could be to target mitochondrial functions and mTOR pathways simultaneously (Figure 7). Based on our results, rapamycin + doxycycline treatment induces autophagy, the consequent sequestration of mitochondria by autophagosomes and final autolysomal mitochondria degradation. In parallel, the doxycycline-inhibited reorganisation of bioenergetic and/or mitochondrial homeostasis result in metabolic catastrophe and anti-tumour effects. We propose that other anti-metabolic combination treatments, which could increase metabolic stress and induce selective autophagy-, mitophagy-dependent cell death, could be utilised in future anti-cancer treatment developments. Additionally, it has to be taken into account that there is a complex metabolic symbiosis in every tumour tissue, which need to be revealed to target adaptation mechanisms in therapy resistance. As we gain more and more knowledge about the importance of metabolic alterations in tumours, the combined application of new or old drug candidates has to be considered as multi-targeted treatment modalities to increase the efficacy of current therapeutic approaches.

## 4. Materials and Methods

### 4.1. Cell Culturing and In Vitro Treatments

For performing in vitro treatments, ten human breast cancer cell lines were selected and applied, these are representing the main hormone and molecular subtypes: (a) luminal A: MCF7—ATCC-HTB-22 and T47D—ATCC-HTB-133; (b) luminal B: BT474—ATCC-HTB-20 and ZR75.1—ATCC-CRL-1500; (c) HER2+: SKBR3—ATCC-HTB-30 and MDA-MB-453—ATCC-HTB-131; triple-negative breast cancers: BT549—ATCC-HTB-122, HS578T—ATCC-HTB-126, MDA-MB-231—ATCC-HTB-26, and MDA-MB-468—ATCC-HTB-132). Other human cell lines with different origins were also involved in specific in vitro experiments (colon carcinoma: HT29—ATCC-HTB-38 and RKO—ATCC-CRL-2577; fibrosarcoma: HT1080—ATCC-CCL-121; glioma/glioblastoma: U87—ATCC-HTB-14, U251—ECACC-09063001, and U373-U—ECACC-08061901; lung adenocarcinoma: H1650—ATCC-CRL-5883 and H1666—ATCC-CRL-5885; melanoma: ATCC-CRL-11147; prostate carcinoma: Du145—ATCCHTB-81). Depending on the cell line culturing conditions, RPMI 1640, DMEM low-glucose or DMEM high-glucose basal media (Biosera – Nuaille, France) were used, supplemented with 10% foetal bovine serum (Biosera), 2 or 4 mM L-glutamine (Biosera) and antibiotics (80 mg/2 mL gentamycin—Sandoz, Basel, Switzerland or 100 UI/mL penicillin-streptomycin—Biosera). Cells were maintained at 37 °C in a humidified atmosphere of 5% CO_2_. 

Cells were seeded into T25 culturing flasks (3–5 × 10^5^ cells/5 mL) for protein expression analyses; 96-well, 6-well plates or chamber slides were used (2–5 × 10^4^ cells/100 µL; 5–8 × 10^5^ cells/3 mL) in Alamar blue (AB) assay or sulforhodamine B (SRB) test and in immune-fluorescent stainings/flow cytometry analyses. As treatment agents, the following were used in monotherapy and combinations: rapamycin (R—mTORC1 inhibitor; 50 ng/mL; Merck-Sigma-Aldrich), Darmstadt, Germany, doxycycline hyclate (D—antibiotic; 10 µM; Merck-Sigma-Aldrich), doxorubicin (Do—chemotherapeutic agent; 50 ng/mL; TEVA, Debrecen, Hungary), chloroquine (Chl—autophagy inhibitor; 50 µM; Merck-Sigma-Aldrich), necrostatin-1 (nec-1—necrosis inhibitor; 50 µM; Merck-Sigma-Aldrich) and Ac-DEVD-CHO (caspase-3 inhibitor; 1 µM; Molecular Probes, Leiden, The Netherlands). The applied concentrations were selected based on our and others’ previous studies.

The combination index (*CI*) calculation method was applied to assess the quantitative evaluation of the effects of the investigated treatment agents. The calculation formula was the following: $CI=\frac{E_{a}+E_{b}}{E_{ab}}\;$, where *E_a_* and *E_b_* were the detected effect of individual mono-treatments and *E_ab_* represents the effect of the combination. Depending on the determined value of *CI*, the cell proliferation influencing effects of the treatment combinations could be characterised as follows: (a) no additional effect: *CI* > 1; (b) additive effect: *CI* = 1; and (c) synergistic effect: *CI* < 1 [61].

### 4.2. In Vitro Proliferation Assays—Alamar Blue and Sulforhodamine B Assays

To monitor the short-time effect (72 or 96 h) of the used treatments on cell growth and cellular metabolic activity, the Alamar blue (AB; 10 µL/well; Thermo Fisher Scientific, Waltham, MA, USA) assay was applied. After a 4-h incubation, the fluorescence was measured with a fluorimeter (Fluoroskan Ascent FL; Labsystems International; Ascent Software – Vantaa, Finland) at 570–590 nm. Sulforhodamine B (SRB) test was used for determining the total protein content of control and treated cells. In brief, cells were fixed by adding trichloroacetic acid (10%; 50 µL/well) for one hour at 4°C; then, the wells were washed with distilled water. After a 15-min incubation with SRB (0.4 m/V%; 50 µL/well) at room temperature, the unbound SRB solution was washed with acetic acid (1%). To solubilise the bound SRB, Tris base solution (10 mM; 150 µL/well) was added to each well. Multiskan MS microplate reader (Labsystems International; Transmit Software – Vantaa, Finland) was used for measuring the absorbance at 570 nm.

To study the long-term effects of in vitro treatments after a 72 h pre-treatment, cells were separated into two groups: Group A—only 72 h treatment course was added and Group B—continuous treatment addition (16 days). Cells were subcultured on every 2nd/3rd day. The difference in cell proliferation was followed and used for determining the initial cell number of the next passage. Changes in cell number were followed with the trypan blue dye exclusion method (cell counting). Growth curves were obtained by calculating the cumulative total cell number based on the method described by *Adami* et al. [62].

### 4.3. Protein Expression Studies: Western Blot, Wes^TM^ Simple and Immunohistochemical Analyses

Protein extracts were prepared from lysed cells (using 50 mM Tris; 10% glycerol; 150 mM NaCl; 1% Nonidet-P40; 10 mM NaF; 1 mM phenylmethylsulfonyl fluoride; 0.5 mM NaVO_3_; pH = 7.5) then quantified/quantitated by Bradford reagent (Bio-Rad, Hercules, CA, USA). To detect the base protein expressions and the treatment-induced alterations of in vitro samples, Western blot or Wes^TM^ Simple (ProteinSimple 004-600; Minneapolis, MN, USA) system was used.

For the Western blot method, equal amounts of proteins were separated using SDS-PAGE gels then transferred to the PVDF membrane (wet blotting system, Bio-Rad). Membranes were incubated with the investigated primary antibodies: anti-RIP1 (1:1000, #3493, Cell Signaling Technologies—CST, Leiden, The Netherlands), anti-p62 (1:1000, ab91526, Abcam, Cambridge, UK), anti-LC3B (1:500, #2775, CST) and anti-β-actin (1:2000, A2228, Merck-Sigma-Aldrich) as loading control. As a secondary antibody, Vectastain Elite ABC HRP Kit (Vector Laboratories, Burlingame, CA, USA) was used. The blots were developed with enhanced chemiluminescence (Thermo Fisher ECL Western Blotting Substrate) and visualised with a C-Digit Blot Scanner (LI-COR Biotechnology, Lincoln, NE, USA). Densitometric analysis was performed using the Image Studio Digits program.

Applying Wes^TM^ Simple method, either Anti-Rabbit Detection Kit (ProteinSimple DM-001) or Anti-Mouse Detection Kit (ProteinSimple DM-002) was selected and run in a 12–230-kDa Separation Module (ProteinSimple SM-W004) regarding the studied mTOR/AKT pathway-related primary antibodies tested (anti-p-mTOR—1:50, #2971, CST; anti-p-(Ser473)-AKT—1:50, #4060, CST; anti-p-(Ser240/244)-S6—1:50, #4858, CST). Anti-β-actin (1:50, #2775, Merck-Sigma-Aldrich) was also used as an initial loading control. The procedure was carried out in accordance with the manufacturer’s instructions. In brief, the 1:50 used primary antibodies determined the applied concentration of cell lysates (0.2–1 µg/µL). After diluting the lysates/samples in Wes^TM^ Sample Buffer (ProteinSimple 042-195), Fluorescent Master Mix (1:4, ProteinSimple PS-FL01-8) was added. Following a 5-min incubation step (95 °C), the Antibody Diluent (ProteinSimple 042-203), the primary and secondary antibodies and the chemiluminescent substrate were pipetted into the Wes^TM^ capillary plate subsequently. The previously described default settings of the measurement were the following: (1) separation at 395 V for 30 min, (2) blocking for 5 min, (3) incubation with primary antibodies for 30 min, (4) incubation with the appropriate secondary antibody for 30 min and (5) chemiluminescence detection (luminol/peroxide) for 15 min [24]. The obtained results were analysed with Compass software (San Jose, CA, USA); in case it was needed, a manual correction was done in the electropherograms.

The original and unadjusted images of Western blot and Wes^TM^ Simple analyses can be found in supplementary documentation (Figures S2 and S3).

### 4.4. Immunostainings

Immunohistochemistry was performed on formalin-fixed and paraffin-embedded tumours. Deparaffinised tissue sections underwent antigen retrieval using citrate buffer (pH 6) in a pressure cooker. After endogenous peroxidase blocking, the specimens were incubated with the investigated primary antibodies (anti-LC3B—1:100, #3868, CST; anti-cleaved caspase-3—1:1000, #99664, CST and anti-TOM20—1:500, #42406, CST) at room temperature. Next, Novolink Polymer Detection System (Novocastra) and DAB staining (Aligent, Santa Clara, CA, USA) were used. Slides were counterstained with haematoxylin. Immunostainings were evaluated and documented by two independent observers using Panoramic Viewer Software (3D Histech). Fluorescent stainings were performed using MitoTracker CMXRos (1:10000, #M7512, Invitrogen) and LC3 (1:400, #3868, CST) or TOM20 (1:500, #42406, CST) immunolabelling (after 4% paraformaldehyde fixation, incubation with primary and subsequently secondary goat anti-rabbit Alexa488 IgG antibodies (1:400, #A27034, Thermo Fisher Scientific) and Hoechst (1:600, #H3570, Invitrogen) stainings were used). The immunostainings were analysed using confocal or fluorescent microscopy (Zeiss LSM780. Jena, Germany—Zen Software, Jena, Germany or Nikon Eclipse E600, Nikon Corporation, Tokyo, Japan—Lucia Cytogenetics Software, Laboratory Imaging, Prague, Czech Republic).

### 4.5. Flow Cytometry Measurements

Flow cytometric measurements were performed to differentiate the two main cell-death types. For detecting necrotic cell death, the incorporation of propidium iodide (PI; 15-min, 1 mg/mL; Merck-Sigma-Aldrich) was determined. Apoptosis measurement and cell cycle detection were carried out by using subG1 analysis as described by [63]. Cell preparation for flow cytometric analysis was the following: cell fixation in 70% ice-cold ethanol, alkalic extraction (200 mM Na_2_HPO_4_, pH 7.4), the addition of RNase (100 µg/mL, Merck-Sigma-Aldrich) and staining with PI (1 mg/mL). Measurements were executed with FACSCalibur flow cytometer (Beckman Coulter—Indianapolis, IN, USA). Ten thousand events were acquired in each sample. Data evaluation was performed using Kaluza software (Beckman Coulter).

### 4.6. In Vitro Fluorogenic Caspase-3 Assay

The EnzChek^®^ Caspase-3 Assay Kit #1 (Molecular Probes) was applied to detect apoptosis by the increase in caspase-3 activity. The procedure was performed in accordance with the manufacturer’s protocol. At least 1 × 10^6^ cells were prepared for each sample. The washed cell pellets were lysed with Cell Lysis Buffer and incubated on ice for 30 min. Supernatants were transferred into wells of 96-well plates. Next, 50 µL 5X Reaction Buffer and 1 µL Z-DEVD-AMC was added to all wells. Samples were incubated (room temperature, dark) for 30 min then measured with a fluorimeter (Fluoroskan Ascent FL, Ascent software). Caspase-3 catalysed the release of AMC, which was detected every 5 min for a 50-min period (355–460 nm).

### 4.7. In Vivo Xenograft Model

2.5 × 10^6^ cells of ZR75.1 cells were injected into the breast region of female SCID mice (8 weeks old) subcutaneously to generate xenografts. After the tumour becomes palpable (3–5 weeks), the mice were randomised into five groups. The treatment protocol was the following: (1) control—saline solution intraperitoneal; (2) Rapamune (Pfizer – Budapest, Hungary; active ingredient: rapamycin) by gavage at 3 mg/kg body weight; (3) doxycycline (Merck-Sigma-Aldrich)—5 mg/kg body weight; (4) rapamycin + doxycycline and (5) doxorubicin—(TEVA – Debrecen, Hungary) intravenously at 2 mg/kg body weight. The treatments were administered three times per week for 3 weeks. The body weight and tumour size of mice were assessed and monitored. Tumour volume was calculated using the following equitation: π/6 × (2×  shorter diameter  +  longer diameter)/3)^3^. At the end of the experiments, the tumour weights of euthanized animals were measured. Then the tumours were removed, fixed in 4% paraformaldehyde and embedded in paraffin for further immunohistochemical stainings.

The in vivo study was carried out in accordance with the approval of the Institutional Animal Care Facility and the Institutional Ethical Review Board (PE/EA/801-7/2020 approval date: 16 September 2020.) with official permissions (PEI/001/1733-2/2015 approval date: 14 October 2015.).

### 4.8. Transmission Electron Microscopy

The samples were fixed in glutaraldehyde and washed three times in 0.1 M cacodylate buffer. Before araldite embedding, the fixed samples were dehydrated with increasing ethanol row and treated with 1% uranyl acetate in 70% ethanol for 1 h at 4 °C. Contrast-staining was used with uranyl acetate and lead (II) nitrate on the ultrathin sections. The samples were studied using a transmission electron microscope (Hitachi H-7600). Full-size images can be found in the Supplementary (Figure S4).

### 4.9. Statistical Analyses

Mean values and standard deviations (SD) were calculated from three independent experiments with three or more parallels (depending on the assays used). Statistical analyses of in vitro and in vivo experiments were performed using PAST 3.05 software. The Student’s t-test was used for performing statistical calculations. *p* ≤ 0.05 was considered statistically significant.

## 5. Conclusions

We show that rapamycin (mTORC1 inhibitor) combined with doxycycline (antibiotic with an off-target effect on mitochondrial protein translation) has considerable tumour growth inhibitory effects in different cells with various origins. Our results highlight that long-term/continuous doxycycline combined with rapamycin significantly decreased or even stopped cell proliferation leading to a degrading different cell culture system, especially in breast cancers. The rapamycin + doxycycline combination had serious tumour growth inhibition in the performed in vivo experiments, as well. According to our morphological studies, the detected tumour growth inhibition could be characterised by an increase of autophagic vacuoles, shrunken/clamped and impaired mitochondrial structures and cellular disintegration without apoptosis and necrosis in tumour mass; a metabolic stress-induced selective autophagy, mitophagy-dependent cell death.

Based on our results, we suggest considering the induction of these metabolic catastrophe-dependent effects in future cancer therapies using rapamycin and/or doxycycline as an anti-metabolic treatment combination. The studied treatment-induced metabolic stress and selective autophagy-, mitophagy-dependent cell death could be helpful in targeting the growth of tumour tissues with complex metabolic symbiosis and developing therapy resistance. Additionally, we propose the application and selection of new or old drug candidates in correlation with characterised metabolic alterations in tumours. Regarding the detected effect of rapamycin + doxycycline co-treatment, other metabolic inhibitor combinations, as multi-targeted treatment modalities, could help increase the efficacy of current therapeutic approaches.

## Data Availability

Not applicable.

## Supplementary Materials

The following are available online at https://www.mdpi.com/article/10.3390/ijms22158019/s1.
