# Supplementary material for: Rapamycin Plus Doxycycline Combination Affects Growth Arrest and Selective Autophagy-Dependent Cell Death in Breast Cancer Cells
Source: Int J Mol Sci. 2021 Jul 27;22(15):8019. doi: 10.3390/ijms22158019 (PMC8347279; doi:10.3390/ijms22158019)
Supplement: Supplementary file 1 [file ijms-22-08019-s001.zip › ijms-1271569-supplementary.pdf]

International Journal of *Molecular Sciences*

MDPI

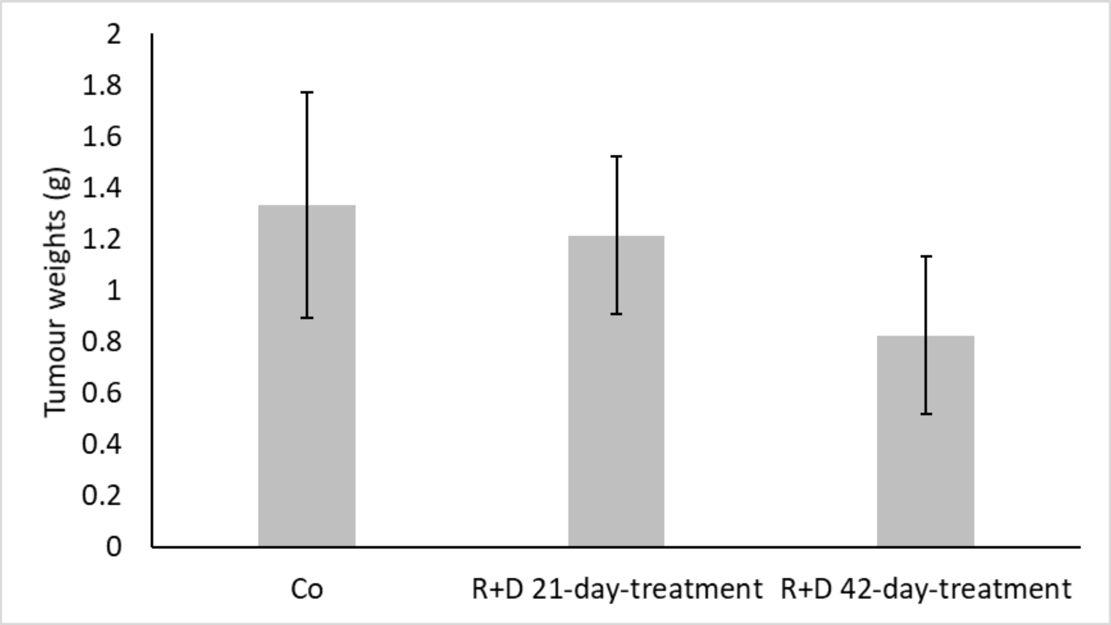

Figure S1 Additional information on *in vivo* tumour growth

The total *in vivo* treatment period was 42 days. At the end of the experiment, the tumour weights were measured. Accordingly, the tumour weights of continuously treated animals were deceased. In contrast, after treatment withdrawal on day 21, the tumours started to grow again.

Figure 2d

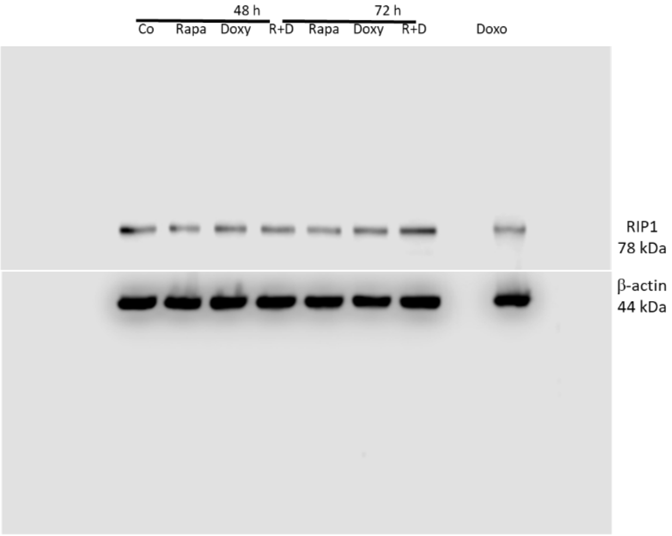

Figure 4b

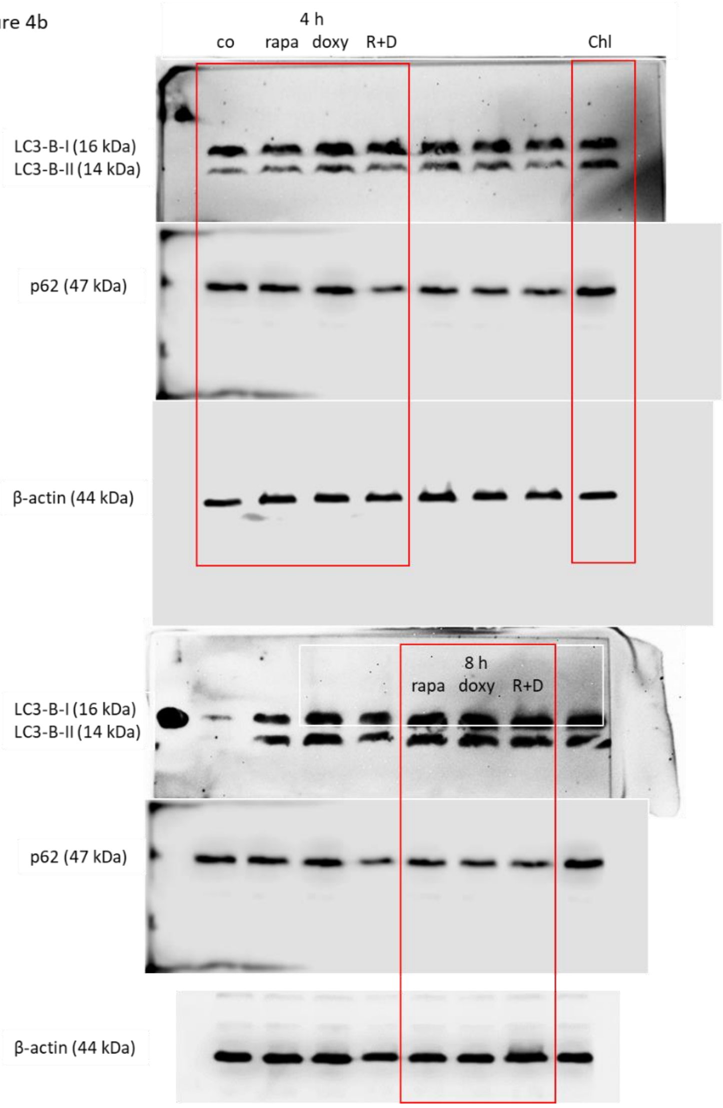

Figure 4b

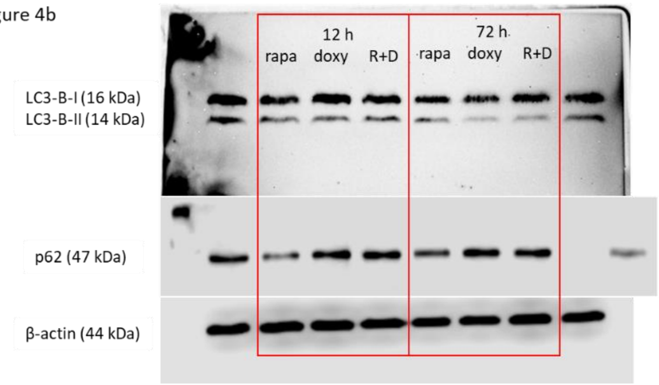

Figure S2 Additional information to Western blots analyses
Original, uncropped images of Western blot gels related to Figure 2d and 4b.

Figure 4a

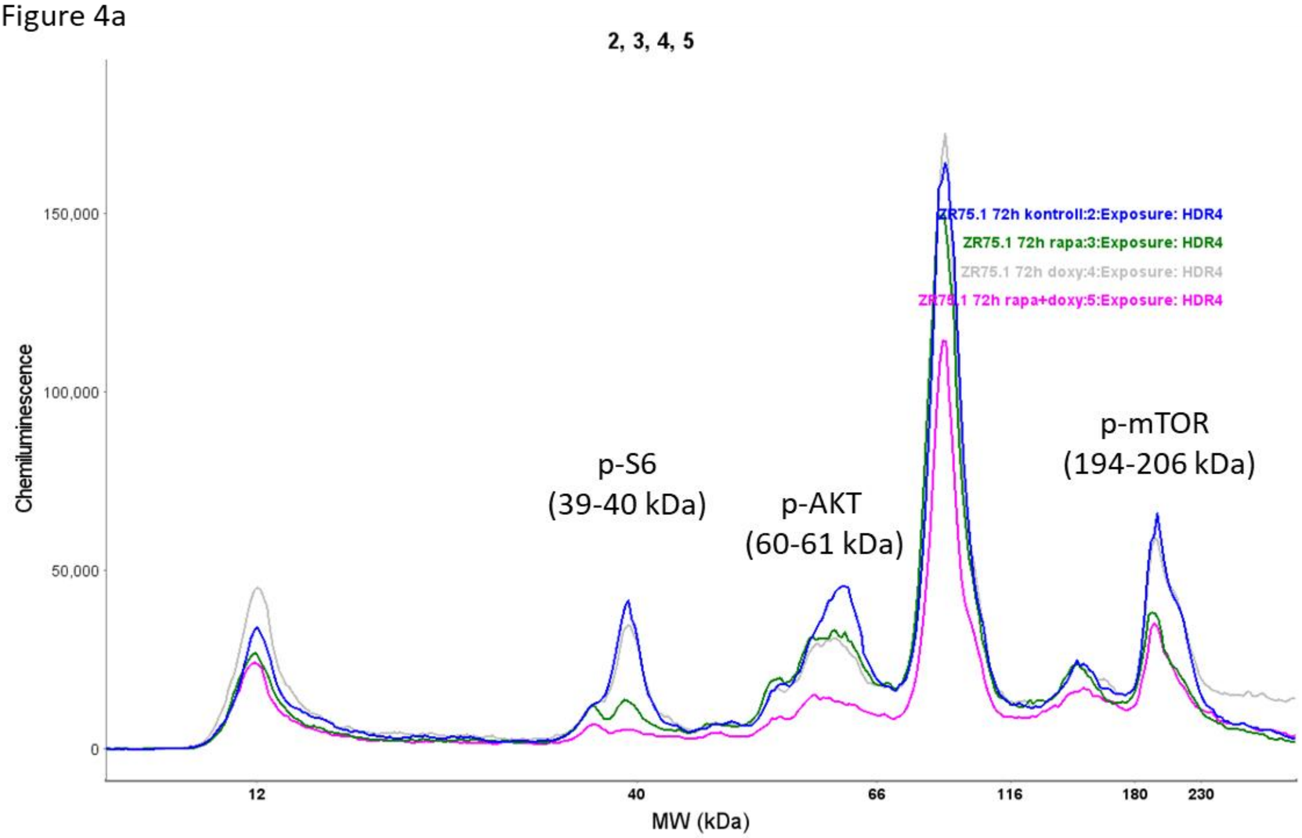

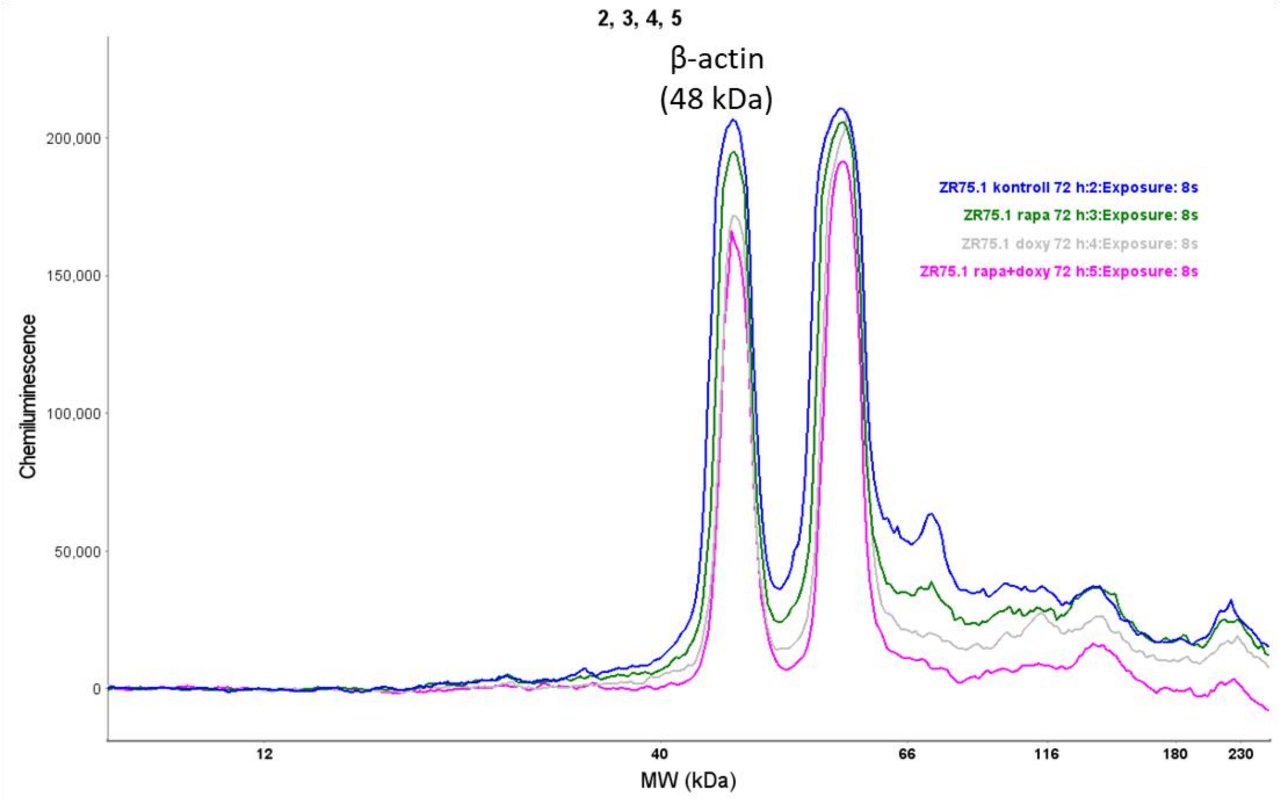

Figure S3 Additional information to WES Simple analyses
Unadjusted elecrtopherograms related to the studied proteins presented in Figure 4a with WES Simple technique.

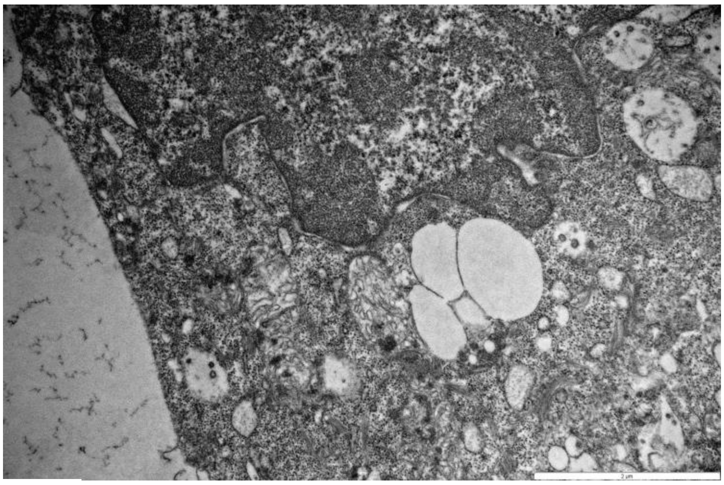

Co

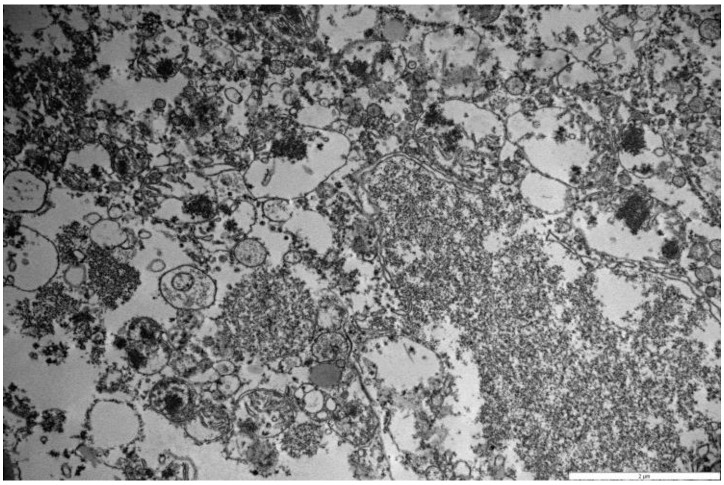

R

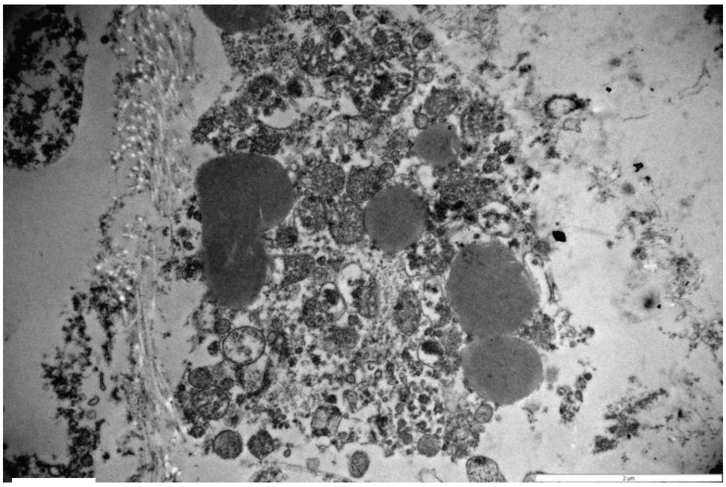

D

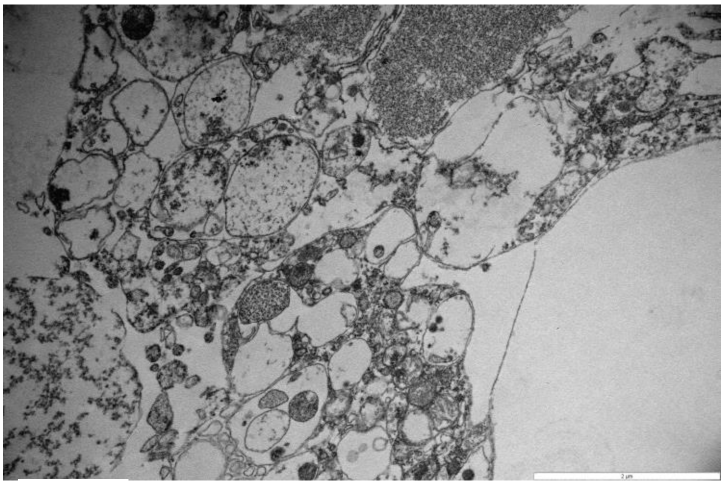

R+D

Figure S4 Full-size image of transmission electron microscopic analyses of long-term rapamycin and doxycycline treated ZR75.1-derived xenografts (magnification 20,000x)
(Co – control; R – rapamycin/Rapamune 3 mg/kg; D – doxycycline 5 mg/kg; R+D – rapamycin/Rapamune + doxycycline combination)
